# Radiotherapy targeting cancer stem cells “awakens” them to induce tumour relapse and metastasis in oral cancer

**DOI:** 10.1038/s41368-020-00087-0

**Published:** 2020-06-24

**Authors:** Yangfan Liu, Miao Yang, Jingjing Luo, Hongmei Zhou

**Affiliations:** 1 State Key Laboratory of Oral Diseases & National Clinical Research Center for Oral Diseases & Department of Oral Medicine, West China Hospital of Stomatology, Sichuan University, Chengdu, China; 2 State Key Laboratory of Oral Diseases & National Clinical Research Center for Oral Diseases & Department of Preventive Dentistry, West China Hospital of Stomatology, Sichuan University, Chengdu, China

**Keywords:** Oral cancer, Cancer stem cells

## Abstract

Radiotherapy is one of the most common treatments for oral cancer. However, in the clinic, recurrence and metastasis of oral cancer occur after radiotherapy, and the underlying mechanism remains unclear. Cancer stem cells (CSCs), considered the “seeds” of cancer, have been confirmed to be in a quiescent state in most established tumours, with their innate radioresistance helping them survive more easily when exposed to radiation than differentiated cancer cells. There is increasing evidence that CSCs play an important role in recurrence and metastasis post-radiotherapy in many cancers. However, little is known about how oral CSCs cause tumour recurrence and metastasis post-radiotherapy. In this review article, we will first summarise methods for the identification of oral CSCs and then focus on the characteristics of a CSC subpopulation induced by radiation, hereafter referred to as “awakened” CSCs, to highlight their response to radiotherapy and potential role in tumour recurrence and metastasis post-radiotherapy as well as potential therapeutics targeting CSCs. In addition, we explore potential therapeutic strategies targeting these “awakened” CSCs to solve the serious clinical challenges of recurrence and metastasis in oral cancer after radiotherapy.

## Introduction

Oral cancer is one of the most commonly diagnosed malignancies in the head and neck region. Currently, the choice to treat oral cancer commonly includes surgery, radiotherapy and/or chemotherapy. Although strategies for treating oral cancer have continued to improve in recent decades, the prognosis of oral cancer remains low, with a long-term disease-free survival of ~50%.^1,2^

Radiotherapy is a critical therapy for cancer patients that causes DNA damage directly via ionising radiation (IR) or by indirectly generating oxidative damage via reactive oxygen species (ROS), thereby leading to cancer cell destruction.^3–5^ In oral cancer, radiotherapy is often selected for early-stage oral cancer patients as a single treatment or for advanced patients in combination with surgery.^6,7^ However, the main challenge in radiotherapy for oral cancer remains locoregional recurrence and/or distant metastasis, which is the major reason that cancer-associated mortality is still high worldwide.^8–10^ As reported, the median overall survival is only 6–9 months for head and neck cancer patients with relapse post-radiotherapy,^11,12^ and 10%–25% of head and neck cancer patients are diagnosed with distant metastasis after radiotherapy;^10^ this situation has a poor prognosis, with a median overall survival of 3–4 months and 1-year-free survival of <5%.^8,13^ Compared to hypopharyngeal and laryngeal cancers, oral cancers have a higher incidence of local relapse and shorter survival when distant metastasis occurs.^14^ Therefore, strategies to address relapse and metastasis of oral cancers post-radiotherapy need to be urgently developed.

It is widely believed that cancer stem cells (CSCs), a small population of cancer cells with self-renewal capacity and multipotent differentiation potential, may be a main cause of cancer relapse and metastasis post-radiotherapy.^15,16^ CSCs are considered to have innately higher radioresistance, invasive capacity and metastatic capacity than their differentiated cancer cell counterparts. Recently, increasing experimental and clinical studies have provided evidence in heterogeneous cancers that a very small number of CSCs surviving after treatment are able to repopulate a tumour at or near the primary cancer site and initiate metastasis development.^17–19^ Complicated cellular and molecular mecha-nisms, such as stemness maintenance, epithelial–mesenchymal transition (EMT) and reactive oxygen species (ROS) production, are involved in the process of CSC initiation and facilitation of cancer recurrence and metastasis after treatment.^20–23^ However, little is known about how CSCs play a role in oral cancer relapse and metastasis after radiation treatment. Herein, we will review the role of CSCs and how CSCs result in oral cancer relapse and metastasis after radiotherapy, as well as the potential mechanisms involved and relevant therapeutic targets.

## Characterisation of oral CSCs

Being able to sort and identify the CSC subpopulation is a prerequisite of further CSC research but is also a major challenge. Stem cell-related markers such as CD44, CD98, CD133 and ALDH1 (aldehyde dehydrogenase 1) are often used for CSC sorting and identification in oral cancer.^24–27^ CD44, a transmembrane glycoprotein, is one of the most common markers used for CSC enrichment^28^ and has been shown to enhance proliferation and survival by sequentially activating the MAPK and P13/AKT pathways.^27,29,30^ CD98^+^ subpopulations have been reported to express high levels of cell cycle and DNA repair genes and to have tumorigenicity in immunodeficient mice.^24^ CD133, a glycoprotein with 5 transmembrane domains, has been identified as a CSC marker in oral cancer; it appears to be involved in angiogenesis and has been shown to be negatively correlated with the prognosis of oral cancer.^26^ ALDH1, an enzyme responsible for detoxification of intracellular aldehydes, has been considered a specific marker of CSCs. ALDH1^+^ cells play a role in the development, metastasis and therapeutic resistance of head and neck squamous cell carcinoma (HNSCC).^31^ Generally, any stem cell-related marker can be used alone or in combination with other markers to sort oral cancer cells with stem cell characteri-stics. However, the specificity of CSC cell markers has been questioned.^32^ Taking CD44 as an example, it has been reported that CD44 is also highly expressed by the majority of normal oral epithelial cells and in oral mucosal tissue.^33,34^ Therefore, it is becoming more common for sorting of CSCs to be enhanced with the use of two or more enrichment markers simultaneously. Some studies indicate that the tumorigenicity of dual-marker-positive cells in vivo is higher than that of single-marker-positive cells, as is their resistance to cancer treatment.^35–37^ However, the lack of one stemness-related marker does not truly mean that cancer cells have no stem cell characteristics because CSCs are heterogeneous.^38–40^ To overcome the limitation of CSC markers in oral cancer, additional confirmation with a function-based enrichment method is a feasible strategy.

The most widely used method of function-based CSC enrichment is the sphere formation assay, in which CSCs can grow non-attached in serum-free medium and form non-adherent 3-dimensional (3D) tumour sphere structures, a characteristic reminiscent of the ability of CSCs to initiate tumours and maintain tumour progression.^41^ Studies have indicated that CSCs that can form spheres retain their stem cell properties for several passages, while most differentiated cells die from anoikis.^42,43^ It has also been reported that sphere formation assays can be used to successfully enrich CSCs from primary oral cancer tissues or oral cancer cell lines.^43^ However, not all primary oral cancer cells can successfully form spheres,^44^ and the purity of CSCs obtained by this method has been questioned, leading to the suggested use of an additional enrichment step before the sphere-forming assay is performed.^34,45^

Additionally, increased drug resistance, an important feature of CSCs, may serve as an alternative factor that can be utilised for CSC enrichment; this increased drug resistance of CSCs is achieved partly due to their high expression of ATP-binding cassette (ABC) transporters, which enable CSCs to efficiently dispel chemotherapeutic agents^44^ and DNA dyes such as Hoechst 33342.^45^ In oral cancer, the CSC subpopulations called side populations (SPs) are identified based on efflux of Hoechst 33342 by flow cytometry or fluorescence-activated cell sorting (FACS), and SP cells have more clonal, invasive and tumorigenic ability than non-side population cells.^46,47^ The advantage of utilising SP sorting as a method to enhance the CSC subpopulation is that it does not require any specific stem cell markers and can be used to enrich CSCs from various tumour tissues and cancer cell lines. However, it is limited to some extent in that the sorted SPs are not homogenous. In addition, the application of this method is technically limited by low specificity, low purity of sorted cells, dye toxicity, variability in dye concentration and dyeing time.^48^

Given that the heterogeneity of oral CSCs is high and the specificity of markers is low, sorting and identifying oral CSCs is still challenging. Both enrichment methods based on stem cell markers and those based on functions have their shortcomings; thus, combining the existing enrichment methods to increase accuracy has been suggested (Table 1).

**Table 1:** Methods for characterising oral CSCs

Methods classification		Biological function	Technique	Refs.
Based on stem cell-related markers	CD44	Cell surface protein, receptor for hyaluronic acid, cell–cell and cell–matrix contacts and migration	Flow cytometry, IHC/ ICC	27–30
	CD98	Cell surface protein, amino acid transport and integrin signalling	Flow cytometry, IHC/ ICC	24
	CD133	Cell surface protein and 5‐transmembrane glycoprotein angiogenesis	Flow cytometry, IHC/ ICC	26
	ALDH1	Retinoic acid production	Aldefluor assay, Flow cytometry	31
Based on CSC functions	Self-renewal	Special property of CSCs to initiate tumour and enhance tumour progression	Sphere formation assay	41–43
	Side population (SP)	ABC transporter-mediated, efflux of endogenous and exogenous DNA dye (Hoechst 33342)	Flow cytometry, FACS	46,47

*IHC* immunohistochemistry; *ICC* immunocytochemistry; *FACS* fluorescence-activated cell sorting

## CSC response to oral cancer radiotherapy

**Fig. 1:**
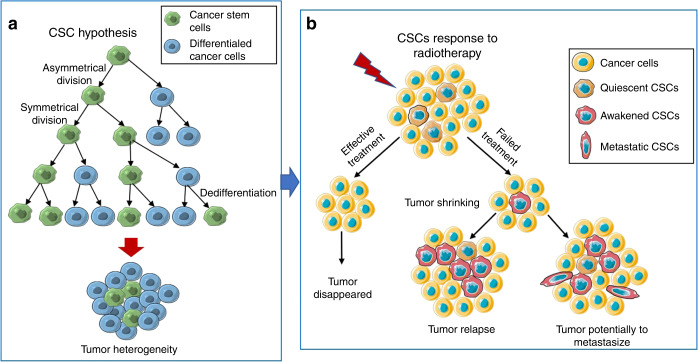
CSC hypothesis and the response of CSCs to radiotherapy. **a** In the CSC hypothesis, the CSC undergoes symmetrical or asymmetric division to give rise to two new CSCs or a differentiated daughter cell and another CSC. Based on the CSC model, the ability to initiate tumorigenesis and generate heterogeneity in primary tumours is fully attributed to the CSC population. **b** In response to radiotherapy, only if all CSCs are eliminated can tumours be permanently eradicated. Moreover, failed radiotherapy can “awaken” quiescent CSCs to enter the cell cycle, leading to tumour relapse, and induce them to transform into metastatic phenotypes, which can eventually result in tumour metastasis

It is widely accepted in the CSC hypothesis that cancer grows as a hierarchy resembling normal tissue, with a small number of cancer stem cells functioning at the top of the hierarchy. Briefly, in this hierarchical CSC model, the ability to initiate tumorigenesis and generate heterogeneous cells in primary tumours is fully encompassed by the CSC population but absent in all differentiated progeny of CSCs (Fig. 1a).^16^ Given this, the response of CSCs to ionizing radiation is critical to the prognosis of cancer patients post-radiotherapy.

Notably, active cell proliferation is a prerequisite for effective chemotherapy and radiotherapy of tumours, and any senescent and quiescent (not only CSCs) cells can be resistant to these therapeutic regimens.^49,50^ This is consistent with the prevailing view that malignant tumours contain dormant cells that are not sensitive to ionising radiation.^51^ It has been reported that even though a large number of differentiated tumour cells are killed by radiotherapy, the dormant cells considered to have some characteristics of CSCs can survive, and these cells are associated with subsequent tumour recurrence or metastasis.^51^ Interestingly, it is generally believed that in advanced cancer, most CSC populations are in a quiescent or dormant state.^52–55^ Studies have demonstrated that approximately one-third of CSCs in glioma and breast cancer cell lines are dormant but enter the cell cycle after radiation, whereas some non-tumorigenic cells (differentiated tumour cells) can become senescent after exposure to radiation.^56,57^ In other words, the quiescent CSC population can be “awakened” by ionising radiation to initiate proliferation and differentiation. Radiotherapy can not only cause dormant CSCs to enter the cell cycle but also induce them to develop a series of malignant phenotypes and carcinogenic metabolism.^58^ Thus, only if all CSCs are eliminated can tumours be permanently eradicated after radiation treatment.^59^

Several studies have shown that radiation treatment preferentially kills non-tumorigenic cells, thus enriching CSCs.^18,60,61^ In addition, radiation can promote reversible transformations between stem and non-stem cells such that new CSCs can be generated from normal and neoplastic non-stem cells,^62–66^ resulting in an increase in the number of CSCs and the coexistence of different types of CSCs, leading to tumour heterogeneity.^67–70^ It has been reported in breast cancer that the absolute number of CSCs is elevated after exposure to ionising radiation, which is not able to be simply explained by the preferential killing of non-tumorigenic cells by ionising radiation.^49^ In addition, it was further confirmed by the same research group that radiation-induced upregulation of the embryonic transcription factors Sox2, Oct4, Klf4 and Nanog in polyploid cells in turn reprogrammes non-tumorigenic cancer cells to acquire CSC properties.^68^ Other scholars also observed that the expression of Sox2, Oct4 and Nanog was upregulated in lymphoma cells with p53 mutations after radiation.^69^ It has also been indicated in two hepatocellular carcinoma cell lines that radiation induces upregulation of Oct3/4 and Sox2, resulting in the acquisition of a CSC phenotype.^67^ Consistent with these results, radiation could induce the dedifferentiation of oral cancer cell lines, leading them to obtain a CSC phenotype.^70^ These findings suggest that differentiated cancer cells acquiring a CSC phenotype is a direct response to radiation rather than a random incidence. Therefore, we propose that in addition to “awakening” quiescent CSC populations, ionizing radiation can also “awaken” some cancer cells with potential stemness, reverting them to a stem-cell-like state.

In summary, it is a major barrier to successful radiotherapy that irradiation can “awaken” cancer stem cells. Current research outcomes suggest that ionising radiation cannot completely kill dormant CSCs because of radioresistance, but it does awaken them, causing them to enter the cell cycling, which further leads to malignant behaviours (Fig. 1b). In addition, ionizing radiation can induce reprogramming of differentiated cancer cells, causing them to dedifferentiate into CSCs, acquiring tumorigenic capabilities in the process.

## CSC-associated relapse and metastasis in oral cancer post-radiotherapy

Radiotherapy remains one of the most common therapeutic approaches for the majority of cancer types. However, it has been reported in some studies that radiotherapy is also able to promote relapse and metastasis.^71–73^ Whether alone or as part of combination treatment, radiotherapy still plays an important role in treating oral cancer at any stage of progression.^6,7^ However, some patients with oral cancer receiving radiotherapy who initially show obvious beneficial effects in terms of shrinkage or eradication of their primary tumours still rapidly develop local tumour recurrence, regional lymph node metastasis or distant lung metastasis.^14,74,75^ It has been reported that compared to other HNSCC tumours, such as hypopharyngeal and laryngeal cancers, oral cancers have a relatively higher recurrence rate postradiotherapy, especially advanced oral cancer, and once tumour metastasis occurs following radiotherapy, the prognosis of patients is extremely poor.^14^

Generally, the failure of radiotherapy for cancers is closely related to various factors, such as the radioresistance of cancer cells and the enhanced invasion and metastasis of tumours. Moreover, the radioresistance of CSCs is being increasingly recognised as a major factor leading to ineffective radiotherapy.^76^ Compared to differentiated cancer cells, undifferentiated CSCs innately have higher radiation resistance, which is possibly because ionising radiation preferentially kills proliferating tumour cells but is ineffective in quiescent CSCs, as they are in a dormant state. In fact, there is abundant evidence supporting that CSCs play a critical role in relapse and metastasis after radiation in several cancer types.^17–19^ Studies on glioma cells and breast cancer cells have found that radiation could only effectively kill non-CSCs, while CSCs survived due to their resistance to radiotherapy and were awakened from their dormant state, increasing rapidly after a period of time post-radiotherapy.^56,57^ It has also been found in breast cancer that the proportion of CSCs increases significantly after ionising radiation, and the CSCs show enhanced proliferation shortly after treatment, which further results in rapid tumour repopulation.^18^ All the findings mentioned here suggest that the surviving CSCs can maintain the ability to proliferate during intervals between radiotherapy applications or after radiotherapy, eventually inducing tumour relapse and resulting in an increased proportion of CSCs that enhance the malignancy of residual tumours. Consistently, findings in hepatocellular carcinoma indicate that compared to non-CSCs, irradiated CSCs are more capable of tumour formation when injected subcutaneously into nude mice.^19^ In addition, it has also been illustrated in non-small-cell lung cancer that cancer cells surviving radiotherapy obtain upregulation of markers of CSCs and mesenchymal cells, as well as the capacity to self-renew and to give rise to differentiated daughters,^17^ further confirming that the cancer cells surviving after radiation have a strongly malignant phenotype.

Although ionizing radiation preferentially kills proliferating tumour cells, nearly half of cancer cells can still survive after radiotherapy.^77^ It has been observed in studies on breast cancer, lymphoma and liver cancer that radiation can lead to upregulation of the expression of embryonic transcription factors by cancer cells, thus inducing the dedifferentiation of cancer cells into CSCs,^67–69^ which is potentially associated with relapse and metastasis post-radiotherapy. Additionally, cells with an IR-induced CSC phenotype have been confirmed to have stronger sphere-forming and tumour-forming abilities in mice,^68^ which means that the number of tumorigenic cells increases after radiation, potentially leading to more rapid tumour recurrence. In addition, it has been confirmed by evidence that CSCs have mesenchymal phenotypes, suggesting their high capacity for migration and invasion.^78–80^ IR can induce non-CSCs to have stronger migration activity, which is closely related to the acquisition of the CSC phenotype and radiation-induced epithelial–mesenchymal transformation (EMT),^58^ which is associated with relapse and metastasis.

For oral cancer, it has recently been revealed that IR can promote the migration of several oral cancer cell lines, including Cal-27, SCC25 and FaDu.^81–83^ Moreover, IR was found to play a significant role in the dedifferentiation of multiple oral cancer cell lines, and 5 days after radiation application, the proportion of CSCs in oral cancer cell lines increased significantly at different IR doses, as assessed by flow cytometry analysis. In addition, the sphere formation assay in this study showed that a single dose of 8 Gy significantly enhanced the sphere-forming ability of oral cancer cell lines.^70^ To draw a preliminary conclusion from the current evidence mentioned above, we believe that the response of oral CSCs to radiotherapy will be found to be similar to that of other CSC types, namely, that oral CSCs can be “awakened” by radiation, further resulting in higher proliferation, migration, invasion and radioresistance, which play a critical role in tumour relapse and metastasis post-radiotherapy.

## Potential mechanism of CSCs in oral cancer relapse and metastasis

**Fig. 2:**
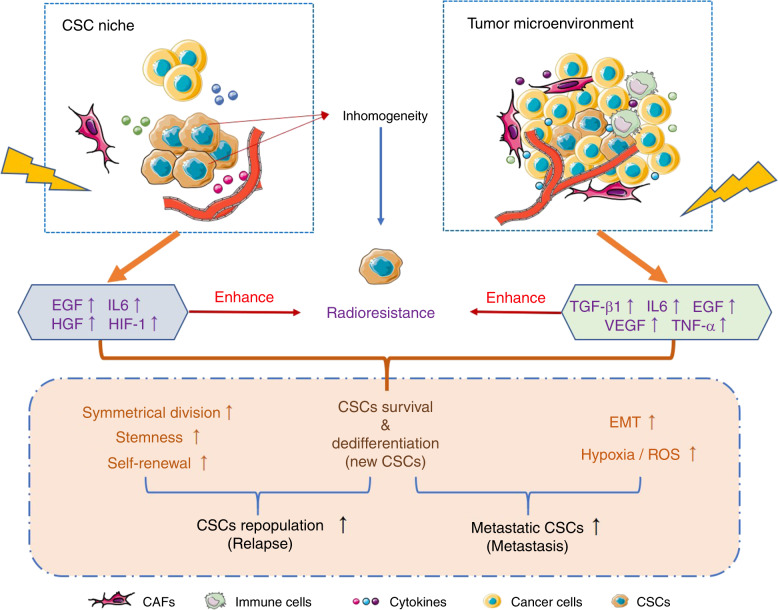
Potential mechanisms by which CSCs induce relapse and metastasis. CSCs have innate radioresistance. Both the CSC niche and tumour microenvironment can enhance the radioresistance of CSCs and support CSC survival during radiotherapy through the expression of multiple cytokines that contribute to increased stemness and self-renewal of CSCs and induce EMT and hypoxic conditions, resulting in higher migration and invasion of CSCs, further leading to tumour recurrence and metastasis

As mentioned above, CSCs are closely related to recurrence and metastasis after radiotherapy for oral cancer, especially the populations of radiation-activated CSCs and those cells that have undergone radiation-induced dedifferentiation to obtain a CSCs phenotype (so called “awakened” CSCs in this review). However, little is known about how “awakened” CSCs result in oral cancer recurrence and metastasis after radiotherapy. Both cancer cells and their surrounding tumour microenvironment play a crucial role in cancer progression, and CSCs initiate tumorigenesis and enhance tumour development. Therefore, radiation-induced changes in CSCs themselves and in their environment should have an effect on recurrence and metastasis post-radiotherapy in oral cancer. Next, we will discuss the potential mechanism by which CSCs cause recurrence and metastasis of oral cancer after radiotherapy. The complex mechanisms are briefly summarised in Fig. 2.

### Innate radioresistance of CSCs

The inherent function of stem cells, permanently providing genetically correct cells to replenish functional tissues, suggests that stem cells have a natural advantage in resisting DNA damage; this resistance aids them in response to radiotherapy, and the radioresistance of these cells is a direct reflection of their inherent ability of DNA repair.^84^ CSCs are considered to have greater radiation resistance than cancer cells, enabling them to survive radiotherapy in various ways, such as controlling the cell cycle, effective DNA repair and modified division patterns.^23,85^ The radioresistance of CSCs due to their innate stemness enables them to survive after radiotherapy, eventually leading to tumour recurrence or metastasis.

Generally, several studies have reported that quiescent CSCs proliferate more slowly than non-CSCs.^52–55^ Studies in glioma and breast cancer have found that one-third of CSCs are dormant and do not enter cell cycling until exposed to ionizing radiation,^49,50^ suggesting a mechanism by which “awakened” CSCs could cause tumour recurrence after radiation. It is also consistent with the idea we discussed above that ionizing radiation is more effective in eradicating rapidly dividing and proliferating cancer cells than dormant or quiescent cells, which helps such dormant cells survive after radiation. Moreover, in both preclinical in vivo models and cancer patients, dormant cells have been confirmed to survive existing therapies, including radiotherapy,^49,50^ which may be the cause of tumour recurrence. In addition to radioresistance due to dormancy, irradiated CSCs also show a stronger ability for DNA repair than differentiated cancer cells. With the detection of gH2AX, a specific DNA damage marker,^86^ it has been reported that in breast cancer, there was no significant difference in the number of gH2AX foci between CSCs and other tumour cells when detected immediately after exposure to ionizing radiation; however, after 48 h, the number of gH2AX foci in CSCs was significantly lower than that in other cancer cells,^87^ indicating that CSCs had a higher capacity for DNA repair. In general, having innate resistance to radiotherapy and high DNA damage repair can synergistically enhance CSC survival during radiotherapy.

### Self-renewal property of CSCs

Self-renewal, a type of cell division specific to stem cells that includes symmetrical and asymmetrical renewal, enables cells to divide indefinitely and retain their differentiation potential.^65^ Generally, CSCs undergo an asymmetrical division, giving rise to a CSC capable of infinite proliferative potential and a differentiated daughter cell with limited division.^88^ However, when tumours are damaged after treatment, such as chemotherapy and radiotherapy, symmetrical CSC division, in which both daughter cells are CSCs that can divide indefinitely, may predominate,^89^ accelerating tumour recurrence. As reported in breast cancer, the number of CD24^−^/CD44^+^ cells increased within a short period of time after radiation accompanied by Notch upregulation.^18^ Findings in glioblastoma showed that CSC enrichment was not only due to the radiation resistance of CSCs but also closely related to the increase in radiation-induced symmetrical division of CSCs, with a potential mechanism that may be related to the activation of the Sonic Hedgehog (SHH), Notch, Wnt and epidermal growth factor receptor (EGFR) signalling pathways.^90^ Radiation-induced Notch and EGFR pathway activation has already been confirmed to enhance self-renewal by increasing symmetric division.^54^

In addition, the high expression of telomerase in CSCs helped maintain their self-renewal ability by playing a role in preventing DNA telomere shortening during cell division, thus allowing cells to proliferate indefinitely,^91^ and promoting EMT of CSCs while still maintaining their stemness.^92,93^ Therefore, the alteration of CSC division post-radiotherapy together with their innate strong self-renewal ability can guarantee the rapid proliferation of surviving CSCs after radiotherapy, further increasing the possibility of tumour relapse.

### CSC-associated epithelial–mesenchymal transition

Epithelial–mesenchymal transition (EMT), a developmental process involved in embryogenesis, wound healing and organ fibrosis,^94,95^ has been confirmed to play an important role in cancer progression and response to cancer treatment.^55^ Briefly, the process of EMT endows epithelial cells with a mesenchymal phenotype that is characterised by the loss of epithelial morphology and markers (including E-cadherin, desmoplakin, muc 1, cytokeratin-18, occludins, claudins and ZO-1) and the acquisition of mesenchymal markers (including N-cadherin, vimentin, fibronectin, vitronectin, α-smooth muscle actin and fibroblast-specific protein 1). Therefore, it is widely believed that cancer cells that have undergone EMT have stronger migration activity than those that retain an epithelial phenotype.^94–96^ There is increasing evidence that CSCs have mesenchymal cell phenotypes,^78–80^ which can ultimately lead to tumour metastasis. It has been found in nasopharyngeal carcinoma that nasopharyngeal carcinoma cell lines surviving radiation treatment show decreased E-cadherin and increased vimentin, resulting in EMT occurrence with strong migration activity.^6^ It has also been demonstrated in other head and neck squamous cancer cell lines that X-ray radiation can promote EMT and enhance migration and invasion.^97^

In addition, radiation-induced EMT of cancer cells has been confirmed in several studies and has been found to be mediated by complex molecular signalling factors, such as TGF-β and ROS.^98–101^ TGF-β1 can be specifically induced by ionising radiation in cancers.^102,103^ Our previous study indicated that radiation could induce increased activation of the TGF-β signalling pathway as well as elevated ROS expression, facilitating oral cancer progression.^104^ It has also been verified through an in vitro study in oral squamous cell carcinoma that TGF-β1 can enhance the CSC phenotype of cancer cells.^105^ Findings in a cervical cancer study showed that TGF-β1 promoted EMT in cancer cells and helped them to obtain a CSC phenotype.^80^ Similarly, in hepatocellular carcinoma, TGF-β1 could achieve the same effect as in cervical cancer by downregulating TP53INP1 through miR-155.^78^ These studies suggest that the radiation-induced TGF-β signalling pathway plays an important role in EMT and obtaining a CSC phenotype, thereby promoting tumour metastasis.

ROS are also involved in TGF-β1-induced EMT in head and neck cancers. In oesophageal adenocarcinoma, TGF-β1 reduces the level of ferritin heavy chain (FHC), which in turn increases the levels of intracellular ROS and activates the p38 MAPK signalling pathway, further inducing EMT. Subsequently, it was further demonstrated that ROS deficiency was able to inhibit the migration of tumour cells.^106^ Moreover, it has been reported in pancreatic cancer that *N*-acetylcysteine (NAC), an antagonist of ROS, can reduce the stemness and EMT phenotype of drug-resistant pancreatic cancer cells.^107^ In addition, inflammatory cytokines, hypoxia-inducing factors and matrix metalloproteinases are all involved in ROS-mediated EMT cascades.^108–110^ These results confirm the key role of ROS in EMT, which indicates that the high concentrations of ROS stimulated by irradiation may promote EMT.

### CSCs and CSCs niches

It has already been confirmed that the anatomically distinct microenvironment containing normal stem cells, called the niche, is composed of a variety of different cells and the extracellular matrix (ECM), which protect normal stem cells and regulates their functions.^111,112^ With increased CSC studies, the “niche” concept has been extended to CSCs, in which CSCs can be supported and induced by the connective tissue matrix and vascular tissue comprising CSC niches.^113,114^ In malignant tumours, CSCs niches promote the divisional dynamics of CSCs, enabling them to produce progenitor cells that further facilitate self-renewal of CSCs and maintain their initial development conditions.^89^ In addition, other cells in CSC niches can function in helping CSCs to metastasise, escape apoptosis, and alter cell division dynamics via activation of various signalling pathways, resulting in cell repopulation.^23,115–117^ It has been reported that nitric oxide produced by endothelial cells in the CSC niche can activate the Notch pathway in glioma, thus promoting self-renewal of CSCs and enhancing their tumorigenic ability in vivo.^118^ In hepatocellular carcinoma, endothelial cells exposed to radiation express elevated IL-4 to activate the ERK and AKT pathways, which promotes the migration and invasion of cancer cells as well as increases the size of the CSC population.^119^ Myofibroblasts in the CSC niche have also been found to activate the Wnt signalling pathway by expressing hepatocyte growth factor, thereby inducing cancer cell dedifferentiation into CSCs and expression of stemness-related genes.^27^

In HNSCC, the majority of CSCs are located perivascularly within a radius of 100 μm, suggesting that a perivascular niche of CSCs exists. In a tumour angiogenesis SCID mouse model, it was observed that specific ablation of tumour-associated endothelial cells resulted in a reduction in head and neck CSCs.^120^ Moreover, the vascular endothelium in oral cancer has also been demonstrated to play an important role in tumour progression; for example, interleukin-6 (IL-6) and epidermal growth factor (EGF) secreted by vascular endothelial cells could promote the stemness of cancer cells in the perivascular niche and improve their vitality, enabling them to escape apoptosis.^115,117^

Similar to the perivascular niche, the CSC niche in response to radiation also provides a hypoxic microenvironment, to further keep CSCs in a quiescent state and to resist radiation-induced oxidative stress,^121^ both of which contribute to CSC radioresistance. While decreased angiogenesis is induced by irradiation in the CSC niche, increased hypoxia associated with growth factor and inflammatory factor expression also occurs, which may accelerate tumour recurrence and the generation of invasive CSCs that contribute to tumour metastasis.^23,116^ In addition, it is widely believed that most cancers have multiple clonal cell types related to CSC subpopulations in the same tumour, leading to tumour heterogeneity. Although it is not yet known whether these heterogeneous CSCs are generated by different niches, some studies suggested that more complex niches could promote the radioresistance of CSCs.^122^

### CSCs and the tumour microenvironment

The tumour microenvironment (TME) refers to the internal environment in which tumour cells are generated and survive, composed of tumour cells themselves; surrounding stromal cells, i.e. fibroblasts and immune and inflammatory cells; intercellular substances; microvessels; and complex molecules in the surrounding area. The TME has been confirmed to play a critical role in mediating the radiation resistance and migration of cancer cells. Ionising radiation can cause a variety of changes in the TME, including increased secretion of various cytokines by stromal cells and recruitment of immune cells, contributing to the self-renewal and stemness maintenance of CSCs as well as associated cancer progression.

Carcinoma-associated fibroblasts (CAFs), as a key component in the TME different from normal fibroblasts, have a modified phenotype via interactions with tumour cells.^123^ It is widely considered in the majority of cancers, including oral cancer, that CAFs could play a positive role in cancer progression through their interaction with cancer cells or paracrine signalling affecting cancer cells, including CSCs.^124–127^ In the study of lung cancer, it was observed that CAFs could activate IGF1R signalling in cancer cells, which further induced the expression of Nanog in cancer cells, contributing to the promotion of the stemness of CSCs, suggesting that CAFs in the CSC niche support cancer stemness.^128^ In addition, many studies have also focused on the effect of radiation on CAF functions and found that radiation can promote the secretion of various cytokines by CAFs with a positive role in cancer progression.^129–132^ It has been observed in colorectal cancer that radiation-activated CAFs promote the metabolic conversion of glutamine through IGF1R activation, which further aggravates cancer progression.^130^ In vitro findings in hepatic carcinomas showed that radiation could induce CAFs to secrete various cytokines, including tumour necrosis factor-α (TNF-α), IL-6, vascular endothelial growth factor (VEGF), epidermal growth factor (EGF), MMP2 and MMP9,^131^ promoting the survival, metastasis and self-renewal of CSCs. It has also been indicated in breast cancer in vivo that pre-irradiation of mice bearing breast cancer increased the number of circulating tumour cells (CTCs, often confirmed to be CSCs) and the incidence of lung metastasis via the elevated expression of cyclo-oxygenase-2 (COX2) and IL-6 in the TME.^133^ Similarly, in squamous cell carcinomas, it has been shown that CAF-secreted TGF-β induced by irradiation can promote the migration and invasion of cancer cells in vitro^71^ and increase the number of CTCs and lung metastasis in breast tumours in situ.^134^

Additionally, radiation can affect the recruitment of immune cells as well as their biological behaviour. After irradiation, tumour-associated macrophages (TAMs) and myeloid-derived suppressor cells (MDSCs) can rapidly infiltrate the irradiated site with increased secretion of cytokines and growth factors related to the stemness of cancer cells, thereby altering the growth and migration of cancer cells.^135–137^ It was found in primary human glioma that the distribution of TAMs in the leading edge of invasive tumours was associated with the presence of CD133^+^ glioma CSCs and that TAMs significantly enhanced the invasiveness of glioma stem cells through paracrine TGF-β1.^138^ In addition, a large number of TAMs were found around CD44^+^ALDH^+^ cells in colon cancer and CD133^+^ALDH^+^ cells in lung cancer, which synergistically activated Sonic Hedgehog pathways in CSCs with IL-6 secretion.^139^

Similar findings in oral cancer patients showed that the level of serum IL-6 in recurrent patients was higher than that in patients with only primary tumours, with further preclinical study showing that the level of IL-6 was crucial for maintaining the self-renewal and tumorigenicity of CSCs in oral cancer.^117^ In addition, TGF-β has also been confirmed to induce oral squamous cell carcinoma cells to take on a CSC phenotype by abolishing FOXO3a.^105^ Although there is an unfortunate lack of studies on the response of oral CSCs to radiotherapy to date, the results in the other cancers mentioned above suggest that the increased secretion of cytokines in the oral cancer TME following radiotherapy may contribute to the recurrence and metastasis of oral cancer post-radiotherapy by enhancing CSC properties.

### CSCs and hypoxia and ROS

Intratumoural hypoxia is an important indicator of poor prognosis in cancer patients and can lead to severe progression, frequent metastasis and resistance to radiotherapy.^140^ It has also been confirmed that hypoxia can enhance the migration of cancer cells,^141^ as well as maintain CSC stemness and promote EMT.^142^

It has been observed that radiation can induce significantly elevated levels of tumour hypoxia inducible factor-1 (HIF-1), which further increases the proportion of hypoxic cells in tumours to regulate tumour radiosensitivity.^143,144^ Importantly, HIF-1 has also been confirmed to be an important mediator in hypoxia, maintaining the stem cell phenotype of CSCs.^145^ It has been reported that under hypoxic conditions, CSCs sorted from FaDu cells, an oropharyngeal cancer cell line, express HIF-1α faster than non-CSCs after radiation. In addition, the radiosensitivity of both CSCs and non-CSCs increased significantly after inhibiting HIF-1α expression even in a hypoxic environment.^146^

It is well known that the accumulation of ROS can result in DNA damage in the majority of tumour cells in response to radiotherapy.^3^ ROS can cause structural damage when they occur within a 2-nanometre range of cellular DNA.^89^ Notably, the ability of ROS to result in DNA damage is attributed to the oxidative stress of irradiated cells; therefore, hypoxic cells have stronger radiation resistance than cells in a normoxic environment.^147^ Therefore, IR-induced additional ROS are less lethal to hypoxic cells and may unexpectedly promote their malignant phenotype. For CSC populations in cancer, it has been found that CSCs can prevent oxidative damage to DNA from ROS by increasing the production of free radical scavengers, further bolstering their inherent radiation resistance.^148^ In addition, CSCs in tumours are more localised to hypoxic areas than to those with normal oxygen concentrations, which will further weaken the lethality of ROS against CSCs and enhance CSC radioresistance.^146^ In summary, radiation-induced ROS production and hypoxic conditions have a close relationship in response to radiotherapy by affecting CSC radioresistance.

## Potential therapeutics targeting CSCS in oral cancer

As reviewed above, relapse and metastasis of oral cancer after radiotherapy remain major challenges. Given the close relationship between CSCs and relapse and metastasis, particularly the “awakened” CSC subpopulations, it is necessary to develop therapeutic strategies targeting CSCs to improve oral cancer prognosis. Although there is limited knowledge on therapeutic strategies targeting oral CSCs, we will briefly discuss current strategies, including treatments targeting CSC markers, CSC self-renewal pathways, CSC-associated EMT and the CSC niche.

### Targeting CSC markers

CSC-targeted therapy can be achieved by directly targeting overexpressed CSC-related molecules. It has been reported that pancreatic cancer patients with high CD44 expression had significantly shorter overall survival than those with low CD44 expression. An anti-CD44 antibody could inhibit the growth and metastasis of transplanted pancreatic tumours in mice, and the anti-CD44 antibody combined with radiotherapy could reduce tumour relapse after radiotherapy.^149^ In preclinical trials in HNSCC patients, it has been shown that combined application of both an anti-CD44v6 monoclonal antibody and maytansinoid (DM1) in patients together with segmentation radiation could significantly improve the control of permanent local tumours.^150^ Additionally, in a previous clinical study of patients with refractory squamous cell carcinoma of the head and neck or oesophagus, it was observed that combined treatment with bivatuzumab, a human monoclonal antibody against CD44v6 and mertansine (DM1), led to better efficacy than monotherapy with either agent, although this clinical study was discontinued after a subject died of toxic epidermal necrolysis.^151^ It is worth noting that the identification of CSC markers should take into account the specificity of the markers. Given that there is a large amount of CD44 in normal oral tissues,^33,34^ it is difficult to use anti-CD44 antibodies targeting CSCs in oral cancer. Additionally, it was found that using lentivirus transfection to silence CD133 in oral squamous cell carcinoma could help decrease the therapy resistance of oral cancer significantly.^26^ Although no clinical studies have been conducted on targeting CSC markers in combination with radiotherapy in oral cancer, the studies mentioned above have provided us with good insight.

### Targeting self-renewal

Their innate self-renewal ability is one of the most important mechanisms of CSCs causing tumour relapse after radiotherapy. Several developmental signalling pathways, such as SHH, Notch and Oct4, are active regulators of autophagy and proliferation of stem cells, providing potential CSC targets to treat cancers. By targeting SHH signalling, the autophagy regulator BMI1 has been proven to play a role in the self-renewal of stem cells.^152^ In addition, BMI1 has been found to be highly expressed by CD133^+^ glioblastoma cells, which promote DNA repair by preferentially activating the DNA double-strand break (DSB) response mechanism; in addition, BMI1 deletion can seriously inhibit the DSB response, resulting in increased sensitivity to radiation, suggesting that pharmacological inhibition of BMI1 combined with radiotherapy may provide an effective means of targeting CSCs.^153^ A similar finding of BMI1 targeting CSCs was observed in CD44^+^ nasopharyngeal carcinoma: when overexpressed BMI1 was knocked out in CD44^+^ cells, DNA damage repair was inhibited, and cell apoptosis increased after radiotherapy.^154^ In addition, in colorectal cancer, downregulation of BMI1 inhibited the self-renewal ability of CSCs, leading to the loss of their tumorigenic potential, while the use of a small molecular inhibitor of BMI1 could cause long-term damage to colorectal xenograft tumours in mice.^155^ Moreover, BMI1 is also highly expressed in CSCs of oral squamous cell carcinoma. BMI1 was able to suppress the ability of oral CSCs to form cell spheres in vitro, and a BMI1 inhibitor could arrest the progression of xenograft tumours in association with a reduced proportion of CSCs.^156^ All these results suggest that strategies targeting CSC self-renewal, such as BMI1 inhibition, have great potential to be combined with radiotherapy in oral cancer for better prognosis.

### Targeting EMT

Radiation-induced EMT may be a key step in the acquisition of the CSC phenotype.^157^ Thus, strategies targeting the EMT-related transformation of cancer cells into CSC-like cells in combination with radiotherapy should offer strong alternatives for improving prognosis in oral cancer. It has been revealed that miR-495 can weaken EMT phenotype acquisition in nasopharyngeal carcinoma cells and enhance their radiosensitivity by downregulating the expression of the cell surface protein GRP78.^158^ Signal transducer and activator of transcription 3 (STAT3), an important transcription factor induced by EMT, has been found to play a role in increasing IR-induced CSCs in pancreatic cancer. In addition, inhibiting the DNA binding activity of STAT3 could reverse its role in the increase in IR-induced CSCs and in turn promote the radiosensitivity of IR-resistant pancreatic cancer cells.^159^ Notably, metformin has been shown to inhibit EMT in a variety of tumours,^160–162^ correspon-dingly contributing to improving the efficiency of radiotherapy in mice xenografted with prostate cancer cells and colorectal cancer cells,^163^ as well as the radiosensitivity of breast cancer and prostate cancer patients, as observed in retrospective studies.^164,165^ In HNSCC, it has been observed that metformin can inhibit cancer progression by acting directly on CSCs through downregulation of cancer stemness signature-associated genes.^166^ It has also been found that metformin can prevent the occurrence of chemical-induced oral cancer and downregulate markers of oral cancer CSCs in mice.^167^ Therefore, targeting the CSC-associated EMT induced by radiation in oral cancer, such as with the use of metformin, is a promising strategy for radiosensitization.

### Targeting the CSC niche

The CSC niche, a complex biological environment that CSCs depend on, has been confirmed to play an important role in the CSC response to radiotherapy. Targeting CSC niches is a potential alternative strategy to damage CSCs during radiotherapy. As mentioned previously, most CSCs in HNSCCs, including oral cancer, are located in the perivascular region, suggesting that the targeted elimination of the CSC perivascular niche may be able to affect the prognosis of oral cancers treated by radiotherapy by reducing nutrient support and the secretion of signalling molecules required by CSCs. Some studies have shown that treating mice bearing cancer with bevacizumab, an anti-angiogenic drug, can result in failure of tumour angiogenesis and significantly reduce CSC numbers as well as tumour growth.^168,169^ In addition, elimination of tumour blood vessels results in increased sensitivity of CSCs to cytotoxic drugs.^170^ Additionally, the specific ablation of tumour-related endothelial cells induced by caspase-9 leads to a reduction in CSCs in head and neck cancers,^120^ suggesting that targeted elimination of the CSC perivascular niche combined with radiotherapy may be a promising oral cancer treatment. In fact, X-ray irradiation of lung adenocarcinoma caused a significant increase in vessel density during tumour formation and increased induction of c-kit phosphorylation in endothelial cells.^171^ Thus, the application of anti-angiogenesis drugs targeting CSC niches in combination with radiotherapy may be a promising therapeutic strategy in cancer, but further studies are needed.

**Fig. 3:**
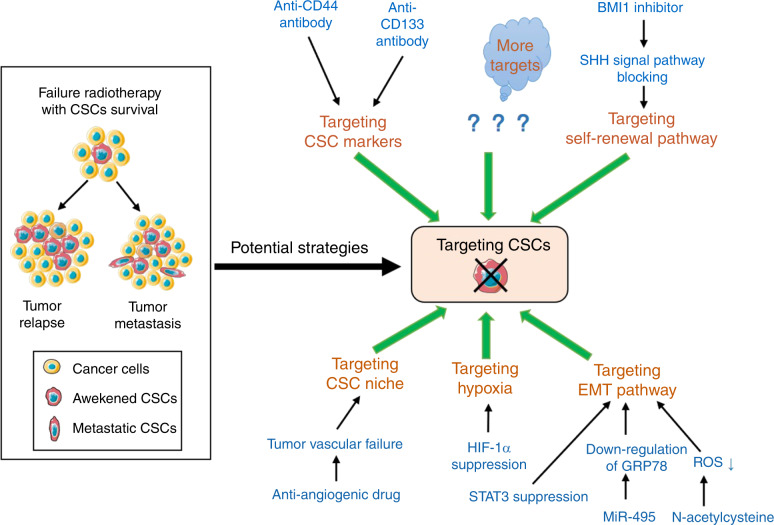
Potential therapeutics targeting oral CSCs. Given that the surviving CSCs post-radiotherapy can lead to relapse and metastasis, a potential strategy targeting oral CSCs needs to be combined with radiotherapy for better efficacy in treating oral cancer. Some examples include treatments targeting CSC markers, CSC self-renewal pathways, CSC niche, CSC-associated EMT and hypoxia

As stated above, there is very limited knowledge to date on targeted therapy for oral CSCs, especially in combination with radiotherapy. However, there is no doubt that new approaches targeting CSCs in combination with radiotherapy may achieve better efficacy to treat oral cancer. Theoretically, it is feasible to directly target CSCs themselves or CSCs niches to achieve radiotherapy sensitisation (Fig. 3). However, the specificity for CSC targets of proposed agents and the toxicity of radiotherapy should be well considered.

## Conclusion

As they are the “seeds” of cancer, the way CSCs respond to treatment is crucial to the prognosis of tumours. Given the results focusing on CSCs and radiation treatment in oral cancer and other cancer types mentioned above, we can preliminarily draw the conclusion that oral CSCs can survive radiotherapy significantly better than non-stem cells, and surviving, quiescent CSCs can be “awakened” by ionising radiation, upregulating cell proliferation, which plays a key role in tumour relapse and metastasis. In addition, oral cancer cells with high malignant potential (often considered to have potential stemness) may also be “awakened” by radiotherapy to dedifferentiate into CSCs or to obtain a CSC phenotype with stemness-related marker expression. As reviewed, the “awakened” CSCs induced by radiation may contribute to oral cancer recurrence and metastasis post-radiotherapy due to their inherent radiation tolerance, DNA repair ability, self-renewal properties and innate mesenchymal phenotype, which endows them with the potential for invasion, and these properties can be reinforced by the CSC niche and tumour microenvironment. However, there is no doubt that our recognition of the role of oral CSCs in tumour recurrence and metastasis post-radiotherapy is still insufficient. More research on how CSCs are “awakened” by radiation and the mechanisms involved in oral cancer relapse and metastasis should be carried out to help explore more potential strategies for targeting oral CSCs, which will ultimately contribute to a better prognosis of oral cancer after radiotherapy.

## References

[1] Bray F (2018). Global cancer statistics 2018: globocan estimates of incidence and mortality worldwide for 36 cancers in 185 countries. CA Cancer J. Clin..

[2] Shahinas J, Hysi D (2018). Methods and risk of bias in molecular marker prognosis studies in oral squamous cell carcinoma. Oral Dis..

[3] Santivasi WL, Xia F (2014). Ionizing radiation-induced DNA damage, response, and repair. Antioxid. Redox Signal..

[4] Feinendegen LE (2002). Reactive oxygen species in cell responses to toxic agents. Hum. Exp. Toxicol..

[5] Surova O, Zhivotovsky B (2013). Various modes of cell death induced by DNA damage. Oncogene.

[6] Su Z (2016). Ionizing radiation promotes advanced malignant traits in nasopharyngeal carcinoma via activation of epithelial-mesenchymal transition and the cancer stem cell phenotype. Oncol. Rep..

[7] Ott S (2018). Radiotherapeutic studies of head and neck cancer-highlights of the 2018 asco annual meeting. HNO.

[8] Russell NS, Bartelink H, Snow GB (1993). Head and neck-cancer. N. Engl. J. Med..

[9] Lambrecht M, Dirix P, Van den Bogaert W, Nuyts S (2009). Incidence of isolated regional recurrence after definitive (chemo-) radiotherapy for head and neck squamous cell carcinoma. Radiother. Oncol..

[10] Schaaij-Visser TBM, Brakenhoff RH, Leemans CR, Heck AJR, Slijper M (2010). Protein biomarker discovery for head and neck cancer. J. Proteom..

[11] Argiris A, Li Y, Forastiere A (2004). Prognostic factors and long-term survivorship in patients with recurrent or metastatic carcinoma of the head and neck. Cancer.

[12] Gold KA, Lee H-Y, Kim ES (2009). Targeted therapies in squamous cell carcinoma of the head and neck. Cancer.

[13] Leon X (2005). A retrospective analysis of the outcome of patients with recurrent and/or metastatic squamous cell carcinoma of the head and neck refractory to a platinum-based chemotherapy. Clin. Oncol..

[14] Leeman JE (2017). Patterns of treatment failure and postrecurrence outcomes among patients with locally advanced head and neck squamous cell carcinoma after chemoradiotherapy using modern radiation techniques. JAMA Oncol..

[15] Vlashi E, Pajonk F (2015). Cancer stem cells, cancer cell plasticity and radiation therapy. Semin. Cancer Biol..

[16] Krause M, Dubrovska A, Linge A, Baumann M (2017). Cancer stem cells: radioresistance, prediction of radiotherapy outcome and specific targets for combined treatments. Adv. Drug Deliv. Rev..

[17] Gomez-Casal, R. et al. Non-small cell lung cancer cells survived ionizing radiation treatment display cancer stem cell and epithelial-mesenchymal transition phenotypes. *Mol. Cancer*10.1186/1476-4598-12-94 (2013). 10.1186/1476-4598-12-94 PMC3751356 23947765

[18] Phillips TM, McBride WH, Pajonk F (2006). The response of CD24(−/low)/CD44(+) breast cancer-initiating cells to radiation. J. Natl Cancer Inst..

[19] Piao LS (2012). CD133(+) liver cancer stem cells modulate radioresistance in human hepatocellular carcinoma. Cancer Lett..

[20] Lyakhovich A, Lleonart ME (2016). Bypassing mechanisms of mitochondria-mediated cancer stem cells resistance to chemo- and radiotherapy. Oxid. Med. Cell. Longev..

[21] Kurth I (2015). Cancer stem cell related markers of radioresistance in head and neck squamous cell carcinoma. Oncotarget.

[22] Chang L (2016). Cancer stem cells and signaling pathways in radioresistance. Oncotarget.

[23] Cojoc M, Mabert K, Muders MH, Dubrovska A (2015). A role for cancer stem cells in therapy resistance: Cellular and molecular mechanisms. Semin. Cancer Biol..

[24] Martens-de Kemp SR (2013). CD98 marks a subpopulation of head and neck squamous cell carcinoma cells with stem cell properties. Stem Cell Res..

[25] Krishnamurthy S, Noer JE (2012). Head and neck cancer stem cells. J. Dent. Res..

[26] Yu C-C, Hu F-W, Yu C-H, Chou M-Y (2016). Targeting CD133 in the enhancement of chemosensitivity in oral squamous cell carcinoma-derived side population cancer stem cells. Head Neck.

[27] Vermeulen L (2010). Wnt activity defines colon cancer stem cells and is regulated by the microenvironment. Nat. Cell Biol..

[28] Prince ME (2007). Identification of a subpopulation of cells with cancer stem cell properties in head and neck squamous cell carcinoma. Proc. Natl Acad. Sci. USA.

[29] Misra S, Toole BP, Ghatak S (2006). Hyaluronan constitutively regulates activation of multiple receptor tyrosine kinases in epithelial and carcinoma cells. J. Biol. Chem..

[30] Ghatak S, Misra S, Toole BP (2005). Hyaluronan constitutively regulates ERBB2 phosphorylation and signaling complex formation in carcinoma cells. J. Biol. Chem..

[31] Chen YC (2009). Aldehyde dehydrogenase 1 is a putative marker for cancer stem cells in head and neck squamous cancer. Biochem. Biophys. Res. Commun..

[32] Kim W-T, Ryu CJ (2017). Cancer stem cell surface markers on normal stem cells. BMB Rep..

[33] Mack, B. & Gires, O. CD44s and CD44v6 expression in head and neck epithelia. *PLoS ONE*10.1371/journal.pone.0003360 (2008). 10.1371/journal.pone.0003360 PMC2566597 18852874

[34] Kaseb HO, Fohrer-Ting H, Lewis DW, Lagasse E, Gollin SM (2016). Identification, expansion and characterization of cancer cells with stem cell properties from head and neck squamous cell carcinomas. Exp. Cell Res..

[35] Han, J., Fujisawa, T., Husain, S. R. & Puri, R. K. Identification and characterization of cancer stem cells in human head and neck squamous cell carcinoma. *BMC Cancer*10.1186/1471-2407-14-173 (2014). 10.1186/1471-2407-14-173 PMC4008349 24612587

[36] Wang J (2017). Identification and characterization of CD133(+) CD44(+) cancer stem cells from human laryngeal squamous cell carcinoma cell lines. J. Cancer.

[37] Keysar, S. B. et al. Regulation of head and neck squamous cancer stem cells by PI3K and SOX2. *J. Natl. Cancer Inst*. 10.1093/jnci/djw189 (2017). 10.1093/jnci/djw189 PMC5025278 27634934

[38] Prasmickaite, L. et al. Aldehyde dehydrogenase (aldh) activity does not select for cells with enhanced aggressive properties in malignant melanoma. *PLoS ONE*10.1371/journal.pone.0010731 (2010). 10.1371/journal.pone.0010731 PMC2874003 20505780

[39] Dittfeld C (2009). CD133 expression is not selective for tumor-initiating or radioresistant cell populations in the CRC cell lines HCT-116. Radiother. Oncol..

[40] Quintana E (2010). Phenotypic heterogeneity among tumorigenic melanoma cells from patients that is reversible and not hierarchically organized. Cancer Cell.

[41] Kim HS, Pearson AT, Nor JE (2016). Isolation and characterization of cancer stem cells from primary head and neck squamous cell carcinoma tumors. Methods Mol. Biol..

[42] Dontu G (2003). In vitro propagation and transcriptional profiling of human mammary stem/progenitor cells. Genes Dev..

[43] Krishnamurthy S, Nor JE (2013). Orosphere assay: a method for propagation of head and neck cancer stem cells. Head Neck.

[44] Lim YC (2011). Cancer stem cell traits in squamospheres derived from primary head and neck squamous cell carcinomas. Oral. Oncol..

[45] Goodell MA, Brose K, Paradis G, Conner AS, Mulligan RC (1996). Isolation and functional properties of murine hematopoietic stem cells that are replicating in vivo. J. Exp. Med..

[46] Tabor MH (2011). Head and neck cancer stem cells: the side population. Laryngoscope.

[47] Loebinger MR (2008). Squamous cell cancers contain a side population of stem-like cells that are made chemosensitive by abc transporter blockade. Br. J. Cancer.

[48] Shen G (2008). Identification of cancer stem-like cells in the C6 glioma cell line and the limitation of current identification methods. In Vitro Cell. Dev. Biol. Anim..

[49] Demicheli R (2005). Breast cancer recurrence dynamics following adjuvant CMF is consistent with tumor dormancy and mastectomy-driven acceleration of the metastatic process. Ann. Oncol..

[50] Kleffel, S. & Schatton, T. in *Systems Biology Of Tumor Dormancy* Vol. 734 (eds H. Enderling, N. Almog, & L. Hlatky) 145–179 (2013).

[51] Skvortsova I, Debbage P, Kumar V, Slwortsov S (2015). Radiation resistance: cancer stem cells (cscs) and their enigmatic pro-survival signaling. Semin. Cancer Biol..

[52] Arnold CR (2014). Rac1 as a multifunctional therapeutic target to prevent and combat cancer metastasis. Oncoscience.

[53] Gao MQ, Choi YP, Kang S, Youn JH, Cho NH (2010). CD24+ cells from hierarchically organized ovarian cancer are enriched in cancer stem cells. Oncogene.

[54] Hittelman WN, Liao Y, Wang L, Milas L (2010). Are cancer stem cells radioresistant?. Future Oncol..

[55] Skvortsov S (2011). Radioresistant head and neck squamous cell carcinoma cells: Intracellular signaling, putative biomarkers for tumor recurrences and possible therapeutic targets. Radiother. Oncol..

[56] Lagadec, C. et al. Survival and self-renewing capacity of breast cancer initiating cells during fractionated radiation treatment. *Breast Cancer Res*. 10.1186/bcr2479 (2010). 10.1186/bcr2479 PMC2880434 20158881

[57] Vlashi E (2009). In vivo imaging, tracking, and targeting of cancer stem cells. J. Natl Cancer Inst..

[58] Lee, S. Y. et al. Induction of metastasis, cancer stem cell phenotype, and oncogenic metabolism in cancer cells by ionizing radiation. *Mol. Cancer*10.1186/s12943-016-0577-4 (2017). 10.1186/s12943-016-0577-4 PMC5282724 28137309

[59] Nassar D, Blanpain C (2016). Cancer stem cells: basic concepts and therapeutic implications. Annu. Rev. Pathol..

[60] Bao S (2006). Glioma stem cells promote radioresistance by preferential activation of the DNA damage response. Nature.

[61] Woodward WA (2007). WNT/beta-catenin mediates radiation resistance of mouse mammary progenitor cells. Proc. Natl Acad. Sci. USA.

[62] Chaffer CL (2011). Normal and neoplastic nonstem cells can spontaneously convert to a stem-like state. Proc. Natl Acad. Sci. USA.

[63] Gupta PB, Chaffer CL, Weinberg RA (2009). Cancer stem cells: mirage or reality?. Nat. Med.

[64] Lathia JD, Mack SC, Mulkearns-Hubert EE, Valentim CLL, Rich JN (2015). Cancer stem cells in glioblastoma. Genes Dev..

[65] Reya T, Morrison SJ, Clarke MF, Weissman IL (2001). Stem cells, cancer, and cancer stem cells. Nature.

[66] Wang T (2015). Cancer stem cell targeted therapy: progress amid controversies. Oncotarget.

[67] Ghisolfi L, Keates AC, Hu X, Lee DK, Li CJ (2012). Ionizing radiation induces stemness in cancer cells. PLoS ONE.

[68] Lagadec C, Vlashi E, Della Donna L, Dekmezian C, Pajonk F (2012). Radiation-induced reprogramming of breast cancer cells. Stem Cells.

[69] Salmina K (2010). Up-regulation of the embryonic self-renewal network through reversible polyploidy in irradiated p53-mutant tumour cells. Exp. Cell Res..

[70] Vlashi E (2016). Radiation-induced dedifferentiation of head and neck cancer cells into cancer stem cells depends on human papillomavirus status. Int. J. Radiat. Oncol. Biol. Phys..

[71] Biswas S (2007). Inhibition of TGF-beta with neutralizing antibodies prevents radiation-induced acceleration of metastatic cancer progression. J. Clin. Invest..

[72] Vonessen CF (1991). Radiation enhancement of the metastasis- a review. Clin. Exp. Metastasis.

[73] Wild-Bode C, Weller M, Rimner A, Dichgans J, Wick W (2001). Sublethal irradiation promotes migration and invasiveness of glioma cells: Implications for radiotherapy of human glioblastoma. Cancer Res..

[74] Roman A (2018). The role of stereotactic body radiotherapy in reirradiation of head and neck cancer recurrence. Crit. Rev. Oncol. Hematol..

[75] Strojan P (2015). Recurrent and second primary squamous cell carcinoma of the head and neck: When and how to reirradiate. Head Neck.

[76] Chi, H. C., Tsai, C. Y., Tsai, M. M., Yeh, C. T. & Lin, K. H. Roles of long noncoding RNAs in recurrence and metastasis of radiotherapy-resistant cancer stem cells. *Int. J. Mol. Sci*. 10.3390/ijms18091903 (2017). 10.3390/ijms18091903 PMC5618552 28872613

[77] Kim BM (2015). Therapeutic implications for overcoming radiation resistance in cancer therapy. Int. J. Mol. Sci..

[78] Liu F, Kong X, Lv L, Gao J (2015). TGF-beta 1 acts through miR-155 to down-regulate TP53INP1 in promoting epithelial-mesenchymal transition and cancer stem cell phenotypes. Cancer Lett..

[79] Moncharmont C (2012). Targeting a cornerstone of radiation resistance: cancer stem cell. Cancer Lett..

[80] Wu L (2017). TGF-beta 1-induced CK17 enhances cancer stem cell-like properties rather than emt in promoting cervical cancer metastasis via the ERK1/2-MZF1 signaling pathway. FEBS J..

[81] Beck C (2012). The kallikrein-kinin-system in head and neck squamous cell carcinoma (hnscc) and its role in tumour survival, invasion, migration and response to radiotherapy. Oral. Oncol..

[82] Eke I, Dickreuter E, Cordes N (2012). Enhanced radiosensitivity of head and neck squamous cell carcinoma cells by beta1 integrin inhibition. Radiother. Oncol..

[83] Pickhard, A. C. et al. Inhibition of radiation induced migration of human head and neck squamous cell carcinoma cells by blocking of egf receptor pathways. *BMC Cancer*10.1186/1471-2407-11-388 (2011). 10.1186/1471-2407-11-388 PMC3224383 21896192

[84] Niwa O (2015). Icrp publication 131: stem cell biology with respect to carcinogenesis aspects of radiological protection. Ann. ICRP.

[85] Ogawa K (2013). Radiotherapy targeting cancer stem cells: current views and future perspectives. Anticancer Res..

[86] Olive, P. L. In *Cytometry, 4th edition: New Developments 75 Methods In Cell Biology* (eds. Z. Darzynkiewicz, M. Roederer, & H. Tanke) 355–373 (2004).

[87] Zhang M, Atkinson RL, Rosen JM (2010). Selective targeting of radiation-resistant tumor-initiating cells. Proc. Natl Acad. Sci. USA.

[88] Yoo YD, Kwon YT (2015). Molecular mechanisms controlling asymmetric and symmetric self-renewal of cancer stem cells. J. Anal. Sci. Technol..

[89] Reid PA, Wilson P, Li Y, Marcu LG, Bezak E (2017). Current understanding of cancer stem cells: Review of their radiobiology and role in head and neck cancers. Head Neck.

[90] Gao X, McDonald JT, Hlatky L, Enderling H (2013). Acute and fractionated irradiation differentially modulate glioma stem cell division kinetics. Cancer Res..

[91] Hannen R, Bartsch JW (2018). Essential roles of telomerase reverse transcriptase htert in cancer stemness and metastasis. FEBS Lett..

[92] Liu Z (2013). Telomerase reverse transcriptase promotes epithelial-mesenchymal transition and stem cell-like traits in cancer cells. Oncogene.

[93] Park J-I (2009). Telomerase modulates Wnt signalling by association with target gene chromatin. Nature.

[94] Lamouille S, Xu J, Derynck R (2014). Molecular mechanisms of epithelial-mesenchymal transition. Nat. Rev. Mol. Cell Biol..

[95] Tsai JH, Yang J (2013). Epithelial-mesenchymal plasticity in carcinoma metastasis. Genes Dev..

[96] De Craene B, Berx G (2013). Regulatory networks defining emt during cancer initiation and progression. Nat. Rev. Cancer.

[97] Wozny, A. S. et al. Ros production and distribution: a new paradigm to explain the differential effects of x-ray and carbon ion irradiation on cancer stem cell migration and invasion. *Cancers*10.3390/cancers11040468 (2019). 10.3390/cancers11040468 PMC6521340 30987217

[98] De Bacco F (2011). Induction of met by ionizing radiation and its role in radioresistance and invasive growth of cancer. J. Natl Cancer Inst..

[99] Kawamoto A (2012). Radiation induces epithelial-mesenchymal transition in colorectal cancer cells. Oncol. Rep..

[100] Moncharmont C (2014). Radiation-enhanced cell migration/invasion process: a review. Crit. Rev. Oncol. Hematol..

[101] Zhang X, Li XY, Zhang N, Yang QF, Moran MS (2011). Low doses ionizing radiation enhances the invasiveness of breast cancer cells by inducing epithelial-mesenchymal transition. Biochem. Biophys. Res. Commun..

[102] Martin M (1997). Coactivation of AP-1 activity and TGF-beta 1 gene expression in the stress response of normal skin cells to ionizing radiation. Oncogene.

[103] O’Malley YX, Zhao WL, Barcellos-Hoff MH, Robbins MEC (1999). Radiation-induced alterations in rat mesangial cell Tgfb1 and Tgfb3 gene expression are not associated with altered secretion of active Tgfb isoforms. Radiat. Res..

[104] Luo J (2019). Smad7 promotes healing of radiotherapy-induced oral mucositis without compromising oral cancer therapy in a xenograft mouse model. Clin. Cancer Res..

[105] Li K (2019). TGF beta induces sternness through non-canonical AKT-FOXO3A axis in oral squamous cell carcinoma. Ebiomedicine.

[106] Zhang K-H (2009). Ferritin heavy chain-mediated iron homeostasis and subsequent increased reactive oxygen species production are essential for epithelial-mesenchymal transition. Cancer Res..

[107] Zhao H (2017). Up-regulation of glycolysis promotes the stemness and emt phenotypes in gemcitabine-resistant pancreatic cancer cells. J. Cell. Mol. Med..

[108] Cannito S (2008). Redox mechanisms switch on hypoxia-dependent epithelial-mesenchymal transition in cancer cells. Carcinogenesis.

[109] Jin Y (2010). Antineoplastic mechanisms of niclosamide in acute myelogenous leukemia stem cells: Inactivation of the NF-kappa b pathway and generation of reactive oxygen species. Cancer Res..

[110] Radisky DC (2005). Rac1b and reactive oxygen species mediate MMP-3-induced emt and genomic instability. Nature.

[111] Moore KA, Lemischka IR (2006). Stem cells and their niches. Science.

[112] Sneddon JB, Werb Z (2007). Location, location, location: the cancer stem cell niche. Cell Stem Cell.

[113] LaBarge MA (2010). The difficulty of targeting cancer stem cell niches. Clin. Cancer Res..

[114] Oskarsson T, Batlle E, Massague J (2014). Metastatic stem cells: sources, niches, and vital pathways. Cell Stem Cell.

[115] Krishnamurthy S (2014). Endothelial interleukin-6 defines the tumorigenic potential of primary human cancer stem cells. Stem Cells.

[116] Kuonen F, Secondini C, Rueegg C (2012). Molecular pathways: emerging pathways mediating growth, invasion, and metastasis of tumors progressing in an irradiated microenvironment. Clin. Cancer Res..

[117] Zhang M (2014). Elevated intrinsic cancer stem cell population in human papillomavirus-associated head and neck squamous cell carcinoma. Cancer.

[118] Charles N (2010). Perivascular nitric oxide activates notch signaling and promotes stem-like character in pdgf-induced glioma cells. Cell Stem Cell.

[119] Kim, S. D. et al. The malignancy of liver cancer cells is increased by IL-4/erk/akt signaling axis activity triggered by irradiated endothelial cells. *J. Radiat. Res.*10.1093/jrr/rraa002 (2020). 10.1093/jrr/rraa002 PMC7299255 32100006

[120] Krishnamurthy S (2010). Endothelial cell-initiated signaling promotes the survival and self-renewal of cancer stem cells. Cancer Res..

[121] Brunner TB, Kunz-Schughart LA, Grosse-Gehling P, Baumann M (2012). Cancer stem cells as a predictive factor in radiotherapy. Semin. Radiat. Oncol..

[122] Peitzsch C, Kurth I, Kunz-Schughart L, Baumann M, Dubrovska A (2013). Discovery of the cancer stem cell related determinants of radioresistance. Radiother. Oncol..

[123] Kalluri R, Zeisberg M (2006). Fibroblasts in cancer. Nat. Rev. Cancer.

[124] Xing F, Saidou J, Watabe K (2010). Cancer associated fibroblasts (cafs) in tumor microenvironment. Front. Biosci. Landmark.

[125] Hu, Y. et al. Fibroblast-derived exosomes contribute to chemoresistance through priming cancer stem cells in colorectal cancer. *PLoS ONE*10.1371/journal.pone.0125625 (2015). 10.1371/journal.pone.0125625 PMC4418721 25938772

[126] Wu, F. et al. Regulation of proliferation and cell cycle by protein regulator of cytokinesis 1 in oral squamous cell carcinoma. *Cell Death Dis*. 10.1038/s41419-018-0618-6 (2018). 10.1038/s41419-018-0618-6 PMC5948203 29752448

[127] Meng W (2014). A systems biology approach identifies effective tumor-stroma common targets for oral squamous cell carcinoma. Cancer Res..

[128] Chen WJ (2014). Cancer-associated fibroblasts regulate the plasticity of lung cancer stemness via paracrine signalling. Nat. Commun..

[129] Li D (2016). Radiation promotes epithelial-to-mesenchymal transition and invasion of pancreatic cancer cell by activating carcinoma-associated fibroblasts. Am. J. Cancer Res..

[130] Tommelein J (2018). Radiotherapy-activated cancer-associated fibroblasts promote tumor progression through paracrine IGF1R activation. Cancer Res..

[131] Zhou L-Y, Wang Z-M, Gao Y-B, Wang L-Y, Zeng Z-C (2012). Stimulation of hepatoma cell invasiveness and metastatic potential by proteins secreted from irradiated nonparenchymal cells. Int. J. Radiat. Oncol. Biol. Phys..

[132] Chargari C, Clemenson C, Martins I, Perfettini JL, Deutsch E (2013). Understanding the functions of tumor stroma in resistance to ionizing radiation: Emerging targets for pharmacological modulation. Drug Resist. Updates.

[133] Kamochi N (2008). Irradiated fibroblast-induced bystander effects on invasive growth of squamous cell carcinoma under cancer-stromal cell interaction. Cancer Sci..

[134] Bouchard G (2013). Pre-irradiation of mouse mammary gland stimulates cancer cell migration and development of lung metastases. Br. J. Cancer.

[135] Condeelis J, Pollard JW (2006). Macrophages: obligate partners for tumor cell migration, invasion, and metastasis. Cell.

[136] Kozin SV (2010). Recruitment of myeloid but not endothelial precursor cells facilitates tumor regrowth after local irradiation. Cancer Res..

[137] Russell, J. S. & Brown, J. M. The irradiated tumor microenvironment: Role of tumor-associated macrophages in vascular recovery. *Front. Physiol*. 10.3389/fphys.2013.00157 (2013). 10.3389/fphys.2013.00157 PMC3713331 23882218

[138] Ye X-z (2012). Tumor-associated microglia/macrophages enhance the invasion of glioma stem-like cells via TGF-beta 1 signaling pathway. J. Immunol..

[139] Jinushi M (2011). Tumor-associated macrophages regulate tumorigenicity and anticancer drug responses of cancer stem/initiating cells. Proc. Natl Acad. Sci. USA.

[140] Brown JM (1999). The hypoxic cell: a target for selective cancer therapy—eighteenth Bruce F. Cain Memorial award lecture. Cancer Res..

[141] Sullivan R, Graham CH (2007). Hypoxia-driven selection of the metastatic phenotype. Cancer Metastasis Rev..

[142] Das B (2008). Hypoxia enhances tumor stemness by increasing the invasive and tumorigenic side population fraction. Stem Cells.

[143] Moeller BJ (2005). Pleiotropic effects of HIF-1 blockade on tumor radiosensitivity. Cancer Cell.

[144] Moeller BJ, Richardson RA, Dewhirst MW (2007). Hypoxia and radiotherapy: opportunities for improved outcomes in cancer treatment. Cancer Metastasis Rev..

[145] Yeung TM, Gandhi SC, Bodmer WF (2011). Hypoxia and lineage specification of cell line-derived colorectal cancer stem cells. Proc. Natl Acad. Sci. USA.

[146] Wozny AS (2017). Differential pattern of HIF-1 alpha expression in hnscc cancer stem cells after carbon ion or photon irradiation: one molecular explanation of the oxygen effect. Br. J. Cancer.

[147] Hall, E. J. & Giaccia, A. J. *Radiobiology for the radiologist*. Vol. 6 (Lippincott Williams & Wilkins, 2006).

[148] Diehn M (2009). Association of reactive oxygen species levels and radioresistance in cancer stem cells. Nature.

[149] Li L (2014). Antibody against CD44s inhibits pancreatic tumor initiation and postradiation recurrence in mice. Gastroenterology.

[150] Gurtner K (2012). Combined treatment of the immunoconjugate bivatuzumab mertansine and fractionated irradiation improves local tumour control in vivo. Radiother. Oncol..

[151] Tijink BM (2006). A phase I dose escalation study with anti-CD44v6 bivatuzumab mertansine in patients with incurable squamous cell carcinoma of the head and neck or esophagus. Clin. Cancer Res..

[152] Leung C (2004). Bmi1 is essential for cerebellar development and is overexpressed in human medulloblastomas. Nature.

[153] Facchino S, Abdouh M, Chatoo W, Bernier G (2010). Bmi1 confers radioresistance to normal and cancerous neural stem cells through recruitment of the DNA damage response machinery. J. Neurosci..

[154] Xu X-H (2014). ShRNA targeting Bmi-1 sensitizes CD44(+) nasopharyngeal cancer stem-like cells to radiotherapy. Oncol. Rep..

[155] Kreso A (2014). Self-renewal as a therapeutic target in human colorectal cancer. Nat. Med..

[156] Hu J (2019). Cancer stem cell self-renewal as a therapeutic target in human oral cancer. Oncogene.

[157] Du, B. & Shim, J. S. Targeting epithelial-mesenchymal transition (EMT) to overcome drug resistance in cancer. *Molecules*10.3390/molecules21070965 (2016). 10.3390/molecules21070965 PMC6273543 27455225

[158] Feng X, Lv W, Wang S, He Q (2018). miR495 enhances the efficacy of radiotherapy by targeting grp78 to regulate emt in nasopharyngeal carcinoma cells. Oncol. Rep..

[159] Wu X (2016). Overcoming chemo/radio-resistance of pancreatic cancer by inhibiting STAT3 signaling. Oncotarget.

[160] Zhao, Z. et al. Metformin inhibits the IL-6-induced epithelial-mesenchymal transition and lung adenocarcinoma growth and metastasis. *PLoS ONE*10.1371/journal.pone.0095884 (2014). 10.1371/journal.pone.0095884 PMC4005743 24789104

[161] Vazquez-Martin A (2010). Metformin regulates breast cancer stem cell ontogeny by transcriptional regulation of the epithelial-mesenchymal transition (emt) status. Cell Cycle.

[162] Qu C (2014). Metformin reverses multidrug resistance and epithelial-mesenchymal transition (EMT) via activating AMP-activated protein kinase (AMPK) in human breast cancer cells. Mol. Cell. Biochem..

[163] Zannella VE (2013). Reprogramming metabolism with metformin improves tumor oxygenation and radiotherapy response. Clin. Cancer Res..

[164] Ferro A (2013). Evaluation of diabetic patients with breast cancer treated with metformin during adjuvant radiotherapy. Int. J. Breast Cancer.

[165] Spratt DE (2013). Metformin and prostate cancer: reduced development of castration-resistant disease and prostate cancer mortality. Eur. Urol..

[166] Wu X (2019). Metformin inhibits progression of head and neck squamous cell carcinoma by acting directly on carcinoma-initiating cells. Cancer Res..

[167] Siddappa G (2017). Curcumin and metformin-mediated chemoprevention of oral cancer is associated with inhibition of cancer stem cells. Mol. Carcinog..

[168] Calabrese C (2007). A perivascular niche for brain tumor stem cells. Cancer Cell.

[169] Yang ZJ, Wechsler-Reya RJ (2007). Hit ‘em where they live: targeting the cancer stem cell niche. Cancer Cell.

[170] Brabletz T, Jung A, Spaderna S, Hlubek F, Kirchner T (2005). Opinion: migrating cancer stem cells - an integrated concept of malignant tumour progression. Nat. Rev. Cancer.

[171] Kamlah F (2011). Comparison of the effects of carbon ion and photo irradiation on the angiogenic response in human lung adenocarcinoma cells. Int. J. Radiat. Oncol. Biol. Phys..

